# Electronic cigarette use and chest pain in US adults: Evidence from the PATH study

**DOI:** 10.18332/tid/175732

**Published:** 2024-01-19

**Authors:** Leili Behrooz, Wubin Xie, Aboli Goghari, Rosemarie Robertson, Aruni Bhatnagar, Andrew Stokes, Naomi M. Hamburg

**Affiliations:** 1 Section of Vascular Biology, Whitaker Cardiovascular Institute, Chobanian and Avedisian School of Medicine, Boston University, Boston, United States; 2 Population and Global Health, Lee Kong Chian School of Medicine, Nanyang Technological University, Singapore, Malaysia; 3 Department of Global Health, School of Public Health, Boston University, Boston, United States; 4 Tobacco Regulation and Addiction Center, American Heart Association, Dallas, United States; 5 Department of Medicine, School of Medicine, University of Louisville, Louisville, United States

**Keywords:** smoking cessation, electronic cigarettes, chest pain

## Abstract

**INTRODUCTION:**

Electronic cigarettes (e-cigarette) were introduced for smoking cessation/reduction but have also become popular among the youth. Although e-cigarettes contain fewer toxins than combustible cigarettes, their long-term cardiovascular and pulmonary effects remain unknown. We aimed to assess the association between self-reported chest pain and e-cigarette use.

**METHODS:**

We analyzed data from the PATH (Population Assessment of Tobacco and Health) study wave 4 (2016–2018) and wave 5 (2018–2019). Based on questionnaires from wave 4, we categorized tobacco use as: 1) non-use, 2) exclusive e-cigarette use, 3) combustible cigarette use, and 4) dual use. Presence of established cardiovascular disease was examined at wave 4, and participants aged >40 years were asked about chest pain during wave 5. We used binary logistic regression models to determine the association between tobacco exposures and self-reported chest pain.

**RESULTS:**

We evaluated a total of 11254 adults. The rates of chest pain were 1518 out of 7055 non-users, 49 from 208 exclusive e-cigarette users, 1192 from 3722 combustible cigarette users, and 99 out of 269 dual users. In the multivariable models adjusted for relevant covariates, combustible cigarette users (adjusted odds ratio, AOR=1.77; 95% CI: 1.56–2.01) and dual users (AOR=2.22; 95% CI: 1.61–3.05) had higher odds of reporting ever having chest pain, as well as having chest pain in the past 30 days. Conversely, exclusive e-cigarette users had similar odds of reporting chest pain compared to non-users (AOR=1.03; 95% CI: 0.69–1.54) and lower odds than combustible and dual users. In sensitivity analyses, categorizing individuals based on their reported history of cardiovascular disease, overall findings were similar.

**CONCLUSIONS:**

Exclusive e-cigarette use is associated with a lower rate of chest pain compared to combustible cigarette use and dual use.

## INTRODUCTION

It is well known that tobacco smoking is a major public health threat and it is the most important preventable risk factor for the development of cardiopulmonary disease^1^. The risk of death from cardiovascular disease (CVD) decreases by about half after one year of smoking cessation^2^. Given the evidence regarding the harmful effects of smoking and the rapid decline in CVD-related mortality risk after quitting, electronic cigarettes (e-cigarettes) were introduced. These tobacco products were marketed as ‘reduced-harm’ products compared to combustible cigarettes^3^. Currently, e-cigarettes are used for smoking cessation and as a smoking reduction aid. Among adult e-cigarette users, a majority of e-cigarette users are current or former combustible cigarette users; however, the value of e-cigarette use for these individuals remains unclear^4-7^.

The cardiovascular effects of e-cigarettes relative to combustible cigarettes remain an important clinical question. Though e-cigarettes do not combust tobacco, they still deliver nicotine and other chemicals that may have adverse effects in the vasculature^6-8^. In clinical epidemiology investigations, the association of e-cigarettes with cardiovascular symptoms remains uncertain. Although the association of e-cigarette use with respiratory-related symptoms, such as wheezing, has been investigated in both cross-sectional and longitudinal designs, the association between e-cigarette use and chest pain has not been well studied^9-12^. Clinical symptoms are integral in getting additional information about patients beyond just diseases. Population Assessment of Tobacco Health (PATH) study is a nationally representative study with longitudinal surveys on tobacco use behavior and its health effects^13^. Because of the longitudinal design of the PATH dataset, it is possible to ascertain participants’ product use patterns over time. Data from the PATH study have shown different associations between CVD and tobacco exposure categories^14^. The most recent survey of the PATH study added questions about chest pain that were asked of adults aged >40 years. Given this knowledge gap, we examined the association of self-reported chest pain with multiple cigarette and e-cigarette use patterns.

## METHODS

### Study population

We used the PATH study data set for our analysis. The most recent wave of data collection, wave 5, ran from December 2018 to November 2019. A four-stage stratified area probability sample design was used to select individuals from a civilian, non-institutionalized population in the US.

**Figure 1:**
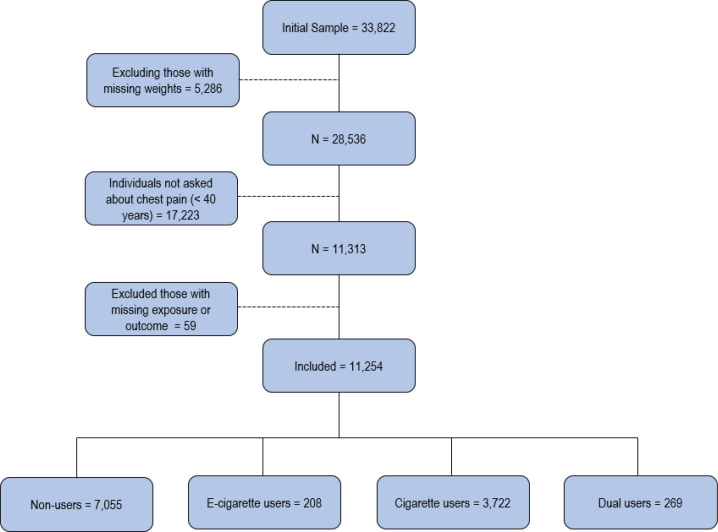
Selection of participants

A survey of chest pain was first introduced in wave 5. The present study used data from wave 4 (exposure wave) and wave 5 (outcome wave). Wave 4 data were collected between December 2016 and January 2018. The study included 11254 adults aged ≥40 years at wave 5 (Figure 1). Because the incidence of chest pain is low among individuals aged <40 years, the PATH questionnaire only asked about chest pain in the cohort of people aged >40 years. Westat’s Institutional Review Board approved the PATH Study protocol and informed consent was obtained from all participants^15^. Our analyses were performed between February to May 2023 and we followed the Strengthening the Reporting of Observational Studies in Epidemiology reporting guideline^16^.

### Current cigarette and e-cigarette use patterns

During wave 4, individuals were asked a series of questions about their cigarette and e-cigarette use behavior. Consistent with previous studies, the main exposure included 4 categories based on current cigarette and e-cigarette use behavior^12,17^. Current cigarette use was defined as having smoked at least 100 cigarettes in a lifetime, and currently smoking cigarettes every day or some days. Current e-cigarette use was defined as having ever used e-cigarettes regularly, and currently using them every day or some days. We then created the four-category exposure: 1) non-use, non-current use of either product; 2) current e-cigarette use, current e-cigarette use, and non-current combustible cigarette use; 3) current cigarette use, current combustible cigarette use, and non-current e-cigarette use; and 4) dual use, current use of both products^10^.

### Outcomes and covariates

Self-reported chest pain at wave 5 was the primary outcome of interest. Two binary outcomes were used: 1) ever had chest pain, chest tightness, or angina; and 2) had chest pain, chest tightness, or angina in the past 30 days.

Informed by current literature^5,18,19^, covariates included age (continuous), sex (female, male), race and ethnicity (non-Hispanic White, non-Hispanic Black, non-Hispanic Other, Hispanic), education level (less than high school, high school/general equivalency development, some college, Bachelor’s degree or higher), body mass index category (<25, 25–29.9, ≥30 kg/m^2^), current use of other combustible tobacco including cigar, pipe, hookah, cigarillo (yes, no), secondhand smoking exposure (yes, no), marijuana use in the past 30 days (yes, no), recreational drug use (yes, no), ever diagnosis for high cholesterol (yes, no), ever diagnosis for hypertension (yes, no) and ever had a history of respiratory disease (yes, no).

### Statistical analysis

Binomial regression models were used to examine the association between the two chest pain outcomes at wave 5, and cigarette and e-cigarette use behavior patterns at wave 4. We tested the interaction between CVD and exposure group with omnibus tests. Our results are from adjusted models and presented as adjusted odds ratios (AORs) and 95% confidence intervals (CIs). Our main model (Model 1) was adjusted for demographics and cardiovascular risk factors including age, sex, race, body mass index, high cholesterol, hypertension and education level. In supplementary analysis, we included two more models. Model 2 was adjusted for age, sex, race and education level. Model 3 was adjusted for covariates in Model 1 plus additional covariates including secondhand smoke, current use of other combustible tobacco, drug use, marijuana use, and history of respiratory disease. All tests were two-sided with a significance level set at p<0.05. All analyses were conducted in STATA version 17 (StataCorp). We accounted for complex survey design using the wave 5 adult single-wave longitudinal weight for the wave 4 cohort sample weights to compensate for different probabilities of selection, non-response, possible deficiencies in the sampling frame, and attrition.

For the main analysis, we used the entire sample including all individuals aged >40 years regardless of history of CVD (heart attack, stroke, heart failure, and any other heart condition). However, because symptoms of chest pain may have different clinical implications between individuals with and those without a history of CVD, we also performed the analysis for both cohorts as two separate groups.

## RESULTS

Among the 11254 adults included in the main analysis, 5410 (53%) of the participants were female, 7261 (70%) were White, 1645 (10%) were Black, 649 (7%) were other races, and 1410 (13%) were Hispanic. The mean age was 56.4 years (SD=11.8) (Table 1). The cohort included 7055 non-users, 208 exclusive e-cigarette users, 3722 combustible cigarette users, and 269 individuals with dual use. The sample without a history of CVD included 9284 participants after excluding 1970 individuals with a history of established CVD at the exposure wave. A majority of exclusive e-cigarette users in the cohort were former cigarette users (92%).

**Table 1:** Characteristics of participants among US adults aged >40 years by exposure type, in the PATH study in a longitudinal setting, data from 2016–2019 (N=11254)

*Characteristics*	*Non-users (N=7055)*		*Exclusive e-cigarette users (N=208)*		*Combustible cigarette users (N=3722)*		*Dual users (N=269)*		*Total (N=11254)*	
	*n (unweighted)*	*% (weighted)*	*n (unweighted)*	*% (weighted)*	*n (unweighted)*	*% (weighted)*	*n (unweighted)*	*% (weighted)*	*n (unweighted)*	*% (weighted)*
**Age** (years), mean (SD)	57.6 (12.1)		54.5 (9.5)		54.5 (9.5)		51.2 (8.4)		56.4 (11.8)	
**Sex**										
Male	3475	46.0	91	48.6	1740	51.1	104	42.4	5410	46.7
Female	3569	54.0	117	51.4	1980	48.9	165	57.6	5831	53.3
**Race**										
Non-Hispanic White	4577	70.6	156	79.0	2327	69.0	201	81.0	7261	70.5
Non-Hispanic Black	953	9.4	17	9.2	647	14.6	28	9.5	1645	10.2
Non-Hispanic Other	386	7.1	18	4.8	226	5.0	19	4.4	649	6.8
Hispanic	956	12.9	15	7.0	424	11.4	15	5.1	1410	12.6
**Education level**										
GED/Lower than high school	1052	13.9	42	19.9	1189	29.9	63	21.2	2346	16.3
High school	1259	23.0	45	29.1	901	30.5	63	28.9	2268	24.2
Some college	2181	28.1	84	34.6	1187	28.6	109	38.0	3561	28.4
Bachelor’s or higher	2542	35.0	37	16.4	428	11.0	32	11.8	3039	31.1
**BMI** (kg/m^2^)										
<25	1678	25.9	62	30.6	1201	33.9	76	25.4	3017	27.1
25–29.9	2420	34.9	64	30.6	1235	33.7	75	29.5	3794	34.6
≥30	2898	39.2	81	38.8	1257	32.4	117	45.1	4353	38.3
**Other combustible**										
No	6546	97.1	191	92.5	3247	88.1	209	77.0	10193	95.6
Yes	490	2.9	17	7.5	465	11.9	59	23.0	1031	4.4
**Secondhand smoke**										
No	5725	84.2	138	65.8	1988	53.1	119	45.6	7970	79.0
Yes	1330	15.8	70	34.2	1734	46.9	150	54.4	3284	21.0
**Drugs**										
No	6960	99.4	198	96.0	3496	95.2	249	93.6	10903	98.7
Yes	68	0.6	9	4.0	196	4.8	19	6.4	292	1.3
**Marijuana**										
Never	5833	89.1	126	60.3	2399	65.3	143	52.2	8501	85.0
Ever, not in past 30 days	695	6.7	42	21.3	569	15.4	55	21.7	1361	8.3
Ever, used in past 30 days	508	4.1	39	18.4	729	19.3	70	26.2	1346	6.7
**Hypercholesteremia**										
No	4226	61.9	114	53.5	2253	63.4	168	65.5	6761	62.1
Yes	2427	38.1	77	46.5	1222	36.6	77	34.5	3803	37.9
**Hypertension**										
No	3662	53.3	121	55.9	1869	52.9	146	55.1	5798	53.3
Yes	3242	46.7	79	44.1	1764	47.1	115	44.9	5200	46.7
**Respiratory disease**										
No	5110	76.7	140	70.2	2287	64.3	140	55.8	7677	74.6
Yes	1747	23.3	59	29.8	1332	35.7	121	44.2	3259	25.4
**Former established cigarette smokers**			193	92.4						

BMI: body mass index.

### Incidence of chest pain across categories

**Figure 2:**
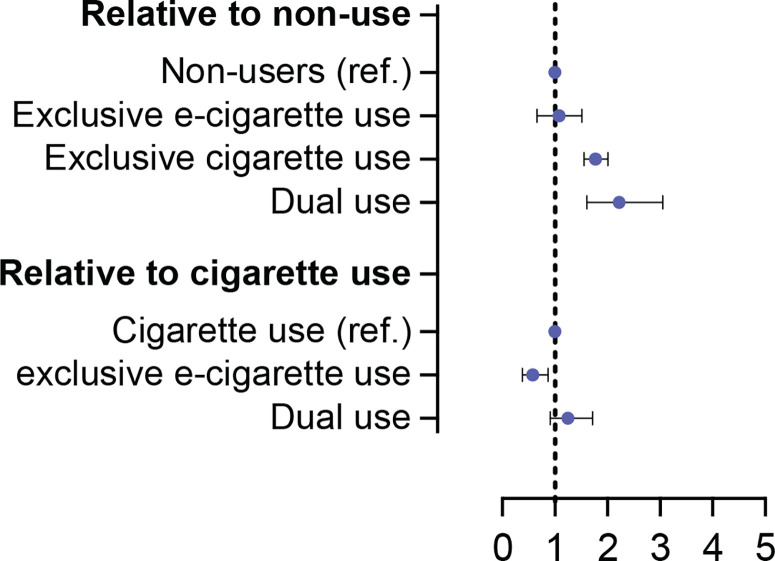
Association of e-cigarette use and chest pain outcomes among US adults, in the PATH study in a longitudinal setting, data from 2016–2019 (N=11254)

At the outcome wave, a total of 2858 participants reported ever having chest pain, and 1450 reported having chest pain in the past 30 days. After adjusting for age, sex, and race/ethnicity, exclusive e-cigarette users had similar rates of ever having chest pain compared to non-users (0.2, 0.19, respectively). Combustible cigarette users and dual users had higher rates of ever having chest pain (0.28, 0.32, respectively) among the four categories. The overall rates were lower for having chest pain in the past 30 days. However, the pattern remained the same with combustible cigarette use and dual use having higher rates (Figure 2).

### Association of chest pain and tobacco use patterns

*Overall sample*

In Model 1, relative to non-use, both combustible cigarette and dual use were associated with significantly higher odds of reporting ever having chest pain (AOR=1.77; 95% CI: 1.56–2.01 and AOR=2.22; 95% CI: 1.61–3.05, respectively) and having chest pain in the past 30 days (AOR=1.98; 95 % CI: 1.68–2.32 and AOR=3.01; 95% CI: 2.06–4.4, respectively). Exclusive e-cigarette use was not associated with chest pain regardless of the recency of the symptom. Compared with combustible cigarette use, exclusive e-cigarette use was associated with significantly lower odds of reporting ever having chest pain (AOR=0.58; 95% CI: 0.39–0.87) as well as chest pain in the past 30 days (AOR=0.51; 95% CI: 0.3–0.85), while dual use had similar odds for both outcomes (Figure 2).

The additional analysis of the association of chest pain and exposure categories in Model 2 showed exclusive e-cigarette users had similar odds of reporting having chest pain ever or having chest pain in the past 30 days compared to non-users. Combustible cigarette users and dual users had significantly elevated odds of ever having chest pain or having chest pain in the past 30 days compared to non-users (Table 2). Similarly, in Model 3, both combustible cigarette use and dual use were associated with significantly higher odds of reporting ever chest pain and having chest pain in the past 30 days compared to non-use, while exclusive e-cigarette use was not associated with chest pain outcomes. Compared with combustible cigarette use, exclusive e-cigarette use was associated with significantly lower odds of reporting chest pain while dual use had similar odds.

**Table 2:** Association of e-cigarette and chest pain outcomes among US adults, in the PATH study in a longitudinal setting, data from 2016–2019 (N=11254)

	*Relative to current non-users*				*Relative to cigarette users*	
	*Current nonusers*	*Dual users AOR (95% CI)*	*Current cigarette users AOR (95% CI)*	*Current vape users AOR (95% CI)*	*Dual users AOR (95% CI)*	*Current vape users AOR (95% CI)*
**Ever had chest pain**						
Model 2	®	2.22 (1.60–3.08)	1.69 (1.50–1.91)	1.06 (0.71–1.58)	1.32 (0.95–1.82)	0.63 (0.42–0.93)
Model 3	®	1.67 (1.22–2.28)	1.47 (1.28–1.68)	0.88 (0.60–1.28)	1.14 (0.84–1.54)	0.60 (0.41–0.87)
**Chest pain in past 30 days**						
Model 2	®	3.06 (2.07–4.52)	1.91 (1.63–2.23)	1.02 (0.61–1.70)	1.60 (1.09–2.36)	0.53 (0.32–0.89)
Model 3	®	2.17 (1.51–3.13)	1.60 (1.34–1.90)	0.85 (0.51–1.41)	1.36 (0.95–1.94)	0.53 (0.32–0.88)

AOR: adjusted odds ratio. Model 2: adjusted for age, sex, race, and education level. Model 3: adjusted for age, sex, race, education level, secondhand smoke, current use of other combustible tobacco, drug use, marijuana use, and history of respiratory disease. ^®^: Reference category.

*Sample without history of CVD*

Among the sample of individuals without CVD, Model 1 showed higher odds of ever having had chest pain among combustible cigarette users (AOR=1.81; 95% CI: 1.56–2.09) and dual users (AOR=1.96; 95% CI: 1.37–2.8) but not for exclusive e-cigarette users (AOR=0.92; 95% CI: 0.57–1.47) compared to non-users. The results were alike for having chest pain in the past 30 days (Table 3).

**Table 3:** Association of e-cigarette and chest pain outcomes among US adults with and without history of CVD, in the PATH study in a longitudinal setting, data from 2016–2019 (N=11254)

	*Sample without history of CVD (N=9284)*				*Sample with history of CVD (N=1970)*			
	*Nonuse*	*Exclusive e-cigarette use AOR (95% CI)*	*Exclusive cigarette use AOR (95% CI)*	*Dual use AOR (95% CI)*	*Nonuse*	*Exclusive e-cigarette use AOR (95% CI)*	*Exclusive cigarette use AOR (95% CI)*	*Dual use AOR (95 % CI)*
**Total**, n	5876	175	3020	213	1179	33	702	56
**Relative to non-use**								
Ever chest pain	®	0.92 (0.57–1.47)	1.81 (1.56–2.09)	1.96 (1.37–2.8)	®	1.41 (0.62–3.18)	1.43 (1.1–1.86)	2.01 (1.02–3.96)
Chest pain in the past 30 days	®	0.87 (0.45–1.66)	2.15 (1.77–2.6)	2.47 (1.63–3.75)	®	1.24 (0.51–3.02)	1.41 (1.04–1.91)	3.33 (1.57–7.05)
**Relative to exclusive cigarette use**								
Ever chest pain		0.51 (0.32–0.81)	®	1.08 (0.76–1.54)		0.98 (0.43–2.23)	®	1.4 (0.71–2.75)
Chest pain in the past 30 days		0.4 (0.21–0.77)	®	1.15 (0.76–1.74)		0.88 (0.36–2.14)	®	2.36 (1.13–4.96)

AOR: adjusted odds ratio; adjusted for age, sex, race, education level, BMI, hypercholesterolemia, and hypertension. ^®^ Reference categories.

*Sample with history of CVD*

In the cohort of individuals with a history of established CVD, we found similar patterns of association between exposure types and chest pain, where combustible cigarette use and dual use were associated with higher odds of ever having chest pain and having chest pain in the past 30 days compared to non-use (Table 3).

## DISCUSSION

This study examined the association of e-cigarette use and chest pain in a large nationally representative cohort of adults aged >40 years in the US. In comparison with non-users, individuals who use combustible cigarettes exclusively or in combination with e-cigarettes have a higher incidence of self-reported chest pain. We observed that exclusive e-cigarette use may be associated with lower risk of chest pain compared to combustible cigarette use. However, dual use of e-cigarettes and combustible cigarettes had similar rates of chest pain relative to combustible cigarette use, suggesting no benefit from a partial switch to e-cigarettes. The pattern of associations was similar between the entire sample and the cohort of individuals without a history of CVD as well as the cohort of individuals with established CVD. Because of the longitudinal design of the PATH study, we have information about participants’ product use in order to do our classification at least one year prior to their report about chest pain. Our data suggest that symptoms that are often attributed to the cardiopulmonary system such as chest pain are less frequent in people who are exclusive e-cigarette users compared to combustible cigarette smokers, but not in people who are dual users.

Our findings extend the prior limited knowledge about e-cigarette use and cardiopulmonary symptoms. With regard to dual users, our results are similar to a cross-sectional report from Health eHeart Study, but differ with respect to e-cigarette use^20^. The Health eHeart study is a cross-sectional study that evaluated the association of cardiopulmonary symptoms including chest pain with different cigarette and e-cigarette use patterns. Their results showed that in comparison with non-users, exclusive e-cigarette users were more likely to report chest pain^20^. Their findings are similar to prior reports in terms of respiratory-related symptoms which found that e-cigarette use is associated with respiratory symptoms such as wheezing^10,12,21^. In both our and the Health eHeart study, the majority of e-cigarette users were former smokers. However, given the longitudinal nature of the PATH dataset, exclusive e-cigarette users were using solely e-cigarettes for at least a year at the time of the outcome wave, whereas this period is unknown in the Health e-Heart study due to its cross-sectional nature. Thus, our observation of lower rates of self-reported chest pain among the exclusive e-cigarette users, as stated in the prior wave, may reflect the benefit of persistent abstinence from combustible cigarette use.

Consistent with the importance of sustained quitting from cigarette use to derive cardiovascular health benefits^22^, we observed that dual users had rates of chest pain similar to combustible cigarette users. Also, prior studies have shown that exposure to toxic substances in dual users is similar to that of combustible cigarette users^17^. Although chest pain is not specific to CVD, it is a key symptom of CVD and can be used for their early diagnosis^23^. With regard to CVD, previous studies have not reported an association between e-cigarette use and CVD; whereas combustible cigarette use and dual use were found to have significantly increased risk of CVD compared to non-use^14,24^. We also showed that dual use was not associated with less chest pain outcomes which is consistent with the known non-linear health effects of combustible cigarette use with CVD, and that only a small number of cigarettes per day is sufficient to increase cardiovascular risk. Given that dual use remains more common than e-cigarette use, it is important to emphasize that this does not appear to be a ‘reduced-harm’ approach^25^. Also, in the analysis of the cohort of participants without a history of CVD, we observed a similar pattern with increased rates of self-reported chest pain among combustible cigarette users and dual users compared to non-users, but not with exclusive e-cigarette use.

In exploratory analyses restricting to individuals with established CVD, which is a small number of individuals, it appears that at least the lack of benefit of dual use is valid, and whether there is a benefit of exclusive e-cigarette use is not clear given the small sample size.

Although the association of e-cigarette use on respiratory-related symptoms, such as wheezing, has been studied with data showing that exclusive use of e-cigarettes may be associated with a higher risk of respiratory symptoms and wheezing^9,10^, there is a lack of data on the association of chest pain with e-cigarette use. Our study provides new information on the relationship between exposure type and self-reported chest pain.

### Limitations

Our study has important limitations. Chest pain may reflect cardiac or non-cardiac etiology and it is not the same as CVD. However, chest pain is a cardinal cardiac symptom that typically requires additional clinical evaluation and may lead to referrals and therefore has important clinical implications. The PATH survey does not include detailed and follow-up questions regarding the characteristics of chest pain and whether participants have had other symptoms that may mimic chest pain such as heartburn or if they have a history of gastroesophageal reflux disease. Also, chest pain was self-reported without physician confirmation. Moreover, the chest pain question was not asked during wave 4 and the results at wave 5 reflect the incidence of chest pain, and we cannot exclude the possibility that they had chest pain at the earlier time as well. We did elect to use the wave 4 data to understand their longitudinal exposure to different tobacco products and that is a clear strength over a cross-sectional design. The number of e-cigarette users in our sample was relatively small and a larger sample size would be needed to examine in detail the health effects of specific types of e-cigarettes. It is important to note that the apparent benefit of e-cigarette use is relevant only to former smokers given the low number of never smokers in the sample. The vast majority of people who were e-cigarette users were former smokers, suggesting that there is a general belief among smokers that switching to e-cigarettes could potentially reduce the cardiovascular and pulmonary risks of smoking combustible cigarettes. The PATH study population includes people from the US, which limits the generalizability of the results to other countries.

## CONCLUSIONS

Our findings from the analysis of this nationally representative cohort of adults are consistent with reduced symptoms of self-reported chest pain from complete replacement with e-cigarettes, but not from dual use with ongoing combustible cigarette smoking. Further work is required to evaluate the range of symptoms attributable to e-cigarettes and their long-term cardiovascular health.

## Data Availability

The data supporting this research cannot be made available for privacy or other reasons.
